# Prognostic Relevance of a Complete Pathologic Response in Liver Transplantation for Hepatocellular Carcinoma

**DOI:** 10.1245/s10434-019-07811-z

**Published:** 2019-09-13

**Authors:** Michał Grąt, Marek Krawczyk, Jan Stypułkowski, Marcin Morawski, Maciej Krasnodębski, Michał Wasilewicz, Zbigniew Lewandowski, Karolina Grąt, Waldemar Patkowski, Krzysztof Zieniewicz

**Affiliations:** 1 Department of General, Transplant and Liver Surgery, Medical University of Warsaw, Warsaw, Poland; 2 Hepatology and Internal Medicine Unit, Department of General, Transplant and Liver Surgery, Medical University of Warsaw, Warsaw, Poland; 3 Department of Epidemiology and Biostatistics, Medical University of Warsaw, Warsaw, Poland; 4 Second Department of Clinical Radiology, Medical University of Warsaw, Warsaw, Poland

## Abstract

**Background:**

A complete pathologic response (CPR) after neoadjuvant treatment is reported to be associated with an exceptionally low risk of recurrence after liver transplantation for hepatocellular carcinoma (HCC). This study aimed to evaluate the prognostic role of CPR in liver transplantation for HCC.

**Methods:**

This retrospective cohort study was based on 222 HCC transplant recipients. Incidence of recurrence and survival at 5 years were the primary and secondary outcome measures, respectively. Competing risk analyses were applied to evaluate recurrence incidence and its predictors. Propensity score matching was performed to compare the outcomes for patients after neoadjuvant treatment with and without CPR.

**Results:**

Neoadjuvant treatment was performed for 127 patients, 32 of whom achieved CPR (25.2%). Comparison of baseline characteristics showed that the patients with CPR were at lowest baseline recurrence risk, followed by treatment-naïve patients and patients without CPR. Adjusted for potential confounders, CPR did not have any significant effects on tumor recurrence. No significant net reclassification improvement was noted after addition of CPR to existing criteria. Neoadjuvant treatment without CPR was associated with increased risk of recurrence in subgroups within the Milan criteria (*p* = 0.016), with alpha-fetoprotein concentration (AFP) model not exceeding 2 points (*p* = 0.021) and within the Warsaw criteria (*p* = 0.007) compared with treatment-naïve patients who were at risk similar to those with CPR. The 5-year incidences of recurrence in propensity score-matched patients with and without CPR were respectively 14.0% and 15.9% (*p* = 0.661), with corresponding survival rates of 73.2% and 67.4%, respectively (*p* = 0.329).

**Conclusions:**

The findings showed that CPR is not independently associated with long-term outcomes after liver transplantation for HCC.

Liver transplantation remains the optimal treatment for selected patients with hepatocellular carcinoma (HCC).^1^ Considering both transplant utility and survival benefit, selection of HCC patients for transplantation should be aimed at keeping the post-transplantation risk of tumor recurrence within acceptable limits. Historically, this has been accomplished through the use of selection criteria based on morphologic features, namely, number and size of lesions, with the well-known Milan criteria as the benchmark for assessment of patient outcomes during more than over 2 decades.^2^–^4^ However, selection of patients based solely on gross morphologic features has resulted in inferior post-transplantation outcomes for HCC patients compared with patients who had non-malignant diseases.^5^ The results of numerous observational studies indicate that these drawbacks may be overcome by combining morphologic factors with surrogates of aggressive biology, most frequently represented by serum alpha-fetoprotein (AFP) concentration.^6^–^10^

The response to neoadjuvant treatments before liver transplantation is increasingly recognized as a marker of tumor biology potentially applicable in more precise selection of patients for transplantation.^11^,^12^ Neoadjuvant treatments including transarterial chemoembolization (TACE), radioembolization, and radiofrequency ablation are commonly used in transplantation centers worldwide, either to decrease the risk of dropout from the waiting list due to tumor progression in patients initially meeting particular selection criteria or to downstage patients beyond them.^13^–^15^

Although both strategies seem efficient, the direct impact of pre-transplantation neoadjuvant therapies on post-transplantation outcomes remains unclear.^16^ Interestingly, although both modified response evaluation criteria in solid tumors (mRECIST) and European Association for the Study of the Liver criteria are based on assessment of induced necrosis, partial tumor necrosis secondary to neoadjuvant therapies has recently been shown to promote lymphatic HCC dissemination.^17^,^18^ In contrast, both a complete radiologic tumor response and particularly a complete pathologic response (CPR) are reported to be associated with favorable outcomes.^19^ However, data on the prognostic role of CPR, even referred to as a surrogate for cancer cure, also are inconsistent.^20^,^21^ Therefore, considering the limited ability to predict tumor response to neoadjuvant treatment and its impact on the risk of tumor recurrence, this study aimed to establish the prognostic relevance of CPR with respect to HCC recurrence in patients undergoing liver transplantation.

## Methods

This retrospective observational study was based on a cohort of 222 consecutive HCC patients after deceased donor liver transplantations performed in the Department of General, Transplant, and Liver Surgery at the Medical University of Warsaw during the period between January 2010 and August 2017. Patients with fibrolamellar HCCs, combined hepatocellular/cholangiocellular carcinomas, and carcinosarcomas were not included in the study.

The study protocol conformed to the ethical guidelines of the 1975 Declaration of Helsinki as reflected in a priori approval by the Medical University of Warsaw human research committee. Due to the retrospective character of the study, informed consent was not required.

A CPR after neoadjuvant therapy, defined as an absence of viable cancer cells on explant pathologic examination, was the primary factor of interest. All histopathologic examinations were performed by pathologists experienced in assessment of hepatobiliary specimens. Neoadjuvant therapies comprised TACE with a mixture of doxorubicin and lipiodol, percutaneous radiofrequency ablation under computed tomography guidance, or a combination of both.

As a routine practice, TACE was performed in cycles of three or two procedures with 4- to 8-week intervals. Patients were individually selected for neoadjuvant treatments during multidisciplinary meetings. In general, patients at presumed high risk of tumor progression beyond Milan criteria were selected for bridging strategy, and those initially beyond Milan criteria were routinely selected for downstaging. Ablative procedures were preferred for patients with solitary lesions up to 3 cm in size, and TACE was performed for larger solitary lesions or multinodular HCCs.

The cumulative incidence of tumor recurrence during the 5-year post-transplantation period was the primary outcome measure. It was based on an end point of tumor recurrence, with death for non-HCC-related causes considered as a competing risk event. Overall survival, the secondary outcome measure, was calculated from the date of transplantation until the patient’s death irrespective of cause and censored 5 years after transplantation or at the last follow-up visit. Data on perioperative management, follow-up protocol, and immunosuppression regimens were provided previously.^22^,^23^

First, baseline characteristics were compared between patients without neoadjuvant treatments, those who had neoadjuvant treatment without CPR, and those who had neoadjuvant treatment with CPR. The three subgroups were compared with respect to post-transplantation outcomes, both in the entire study cohort and after stratification for the initial risk of tumor recurrence according to Milan criteria, total tumor volume (TTV)/AFP criteria, Metroticket 2.0 criteria, French AFP model, and Warsaw criteria.

The potential prognostic significance of CPR also was evaluated in multivariable analyses adjusted for tumor size, tumor number, last pre-transplant AFP, Metroticket 2.0 model, AFP model, and selected selection criteria. To adjust for differences in baseline risk of tumor recurrence, patients with CPR were additionally matched with those not achieving CPR, and post-transplantation outcomes were compared between the two matched cohorts.

Quantitative and qualitative data were presented respectively as medians with interquartile ranges and as numbers with frequencies. Intergroup comparisons were performed using the Mann-Whitney *U* test for quantitative variables and the Chi square test or Fisher’s exact test for qualitative variables. The cumulative incidence of tumor recurrence and its predictors were analyzed using competing risk regression according to Fine and Gray.^24^–^26^ Overall survival estimates were calculated using the Kaplan–Meier method. The log-rank test was used for comparisons of survival curves. The reverse Kaplan–Meier method was used to establish the median duration of follow-up evaluation. Risk factors for impaired overall survival were analyzed using Cox proportional hazards regression. Predictors of CPR were established using logistic regression models.

The potential relevance of CPR for improved prediction of tumor recurrence and patient mortality was evaluated using the Bayesian information criterion (BIC), which provides a measure for choosing a better prognostic model, with lower values indicating improved efficacy. A difference in BIC between the analyzed model and the baseline model (∆BIC) of more than 2 was chosen as the cutoff for omitting the former from further consideration.^26^

Net reclassification improvement (NRI) for adding CPR to the existing selection criteria in prediction of tumor recurrence was established after exclusion of patients with a follow-up period shorter than 2 years. The NRI provides a measure of model predictive performance with respect to improvement of correct reclassification of patients with events and those without events.^27^

Propensity score matching was performed based on the results of logistic regression analyses using the nearest neighbor method in a 1:1 ratio. Hazard ratios (HRs), competing risk regression coefficients (*β*), and NRIs are presented with 95% confidence intervals (CIs). The level of statistical significance was set at 0.05. All *p* values were two-sided. Statistical analyses were computed in STATISTICA version 13.1 (Dell Inc, Tulsa, OK, USA) and R version 3.5.0 (The R Foundation for Statistical Computing, Vienna, Austria).

## Results

Neoadjuvant treatment was performed for 127 (57.2%) of the 222 patients in the study cohort. This included TACE alone for 88 patients (39.6%), RFA alone forn 21 patients (9.5%), and a combination of TACE and RFA for 18 patients (8.1%). Overall, explant pathology showed CPR for 32 (25.2%) of 127 patients after neoadjuvant treatment, including 21 patients after TACE alone (23.9%), 6 patients after RFA alone (28.6%), and 5 patients after the TACE and RFA combination (27.8%; *p* = 0.872).

Compared with the patients who received no neoadjuvant treatment, the patients who underwent neoadjuvant treatment without CPR were older (*p* = 0.026) and had a lower model for end-stage liver disease (MELD) score (*p* < 0.001), larger tumors (*p* < 0.001), a greater total tumor volume (*p* < 0.001), a higher AFP model (*p* = 0.009), a higher Metroticket 2.0 model (*p* = 0.003), and a nonsignificantly higher AFP (*p* = 0.071). These patients also were more often beyond the Milan criteria (*p* = 0.003), the AFP model cutoff of 2 points (*p* = 0.032), and the Warsaw criteria (*p* = 0.019).

In contrast, the patients who underwent neoadjuvant treatment with CPR had fewer tumors (*p* = 0.010) and a lower Metroticket 2.0 model (*p* < 0.001). These patients also were more often within both the Milan criteria (*p* = 0.039) and the AFP model cutoff of 2 points (*p* = 0.042), and uniformly within the limits of Metroticket 2.0 (*p* = 0.001) and the Warsaw criteria (*p* = 0.001) compared with the treatment-naïve patients (Table 1).

**Table 1:** Baseline characteristics of patients with hepatocellular carcinoma (HCC) who had liver transplantation not preceded by neoadjuvant treatment and those who had prior neoadjuvant treatment with and without a complete pathologic response (CPR)

Factors	No neoadjuvant treatment (reference) (*n* = 95)	Neoadjuvant treatment without CPR (*n* = 95)		Neoadjuvant treatment with CPR (*n* = 32)	
	*N* (%) or median (IQR)	*n* (%) or median (IQR)	*p* value	*n* (%) or median (IQR)	*p* value
Recipient sex (male)	72 (75.8)	71 (74.7)	> 0.999	25 (78.1)	> 0.999
Recipient age (years)	57 (52–61)	58 (55–63)	0.026	57 (56–62)	0.400
MELD (points)	11.5 (9.0–15.0)	9 (8–12)	< 0.001	9.5 (8.0–11.5)	0.011
Hepatitis C virus	71 (74.7)	68 (71.6)	0.744	19 (59.4)	0.117
Hepatitis B virus	39 (41.1)	42 (44.2)	0.769	18 (56.3)	0.154
No. of tumors	1 (1–3)	2 (1–3)	0.220	1 (1–1)	0.010
Size of largest tumor (cm)	2.5 (1.5–3.5)	3.5 (2.5–5.0)	< 0.001	3.0 (2.0–4.5)	0.122
Total tumor volume (cm^3^)	12.2 (2.1–33.5)	32.9 (8.7–101.3)	< 0.001	14.1 (4.2–47.7)	0.324
Last pre-transplant AFP (ng/mL)	10.9 (4.6–65.2)	19.8 (6.1–158.1)	0.071	7.8 (4.4–15.7)	0.187
Microvascular invasion	21 (22.3)	28 (29.5)	0.320	–	–
Poor tumor differentiation	11 (11.6)	11 (11.6)	> 0.999	–	–
Within Milan criteria^a^	65 (68.4)	44 (46.3)	0.003	28 (87.5)	0.039
AFP model (points)	1 (0–2)	1 (0–3)	0.009	1 (0–1)	0.367
AFP model ≤ 2 points	77 (81.1)	63 (66.3)	0.032	31 (96.9)	0.042
Metroticket 2.0 (points)	1.8 (1.4–2.8)	2.4 (1.8–3.3)	0.003	0.7 (0.5–1.0)	< 0.001
Within Metroticket 2.0 criteria^b^	73 (76.8)	62 (65.3)	0.109	32 (100.0)	0.001
Within TTV/AFP criteria^c^	81 (85.3)	73 (77.7)	0.195	31 (96.9)	0.113
Within Warsaw criteria^d^	73 (76.8)	57 (60.0)	0.019	32 (100.0)	0.001
Donor age (years)	50 (42–60)	52 (39–61)	0.570	57 (42–63)	0.199
Donor sex (male)	63 (66.3)	55 (57.9)	0.295	17 (53.1)	0.207

*IQR* interquartile range, *MELD* model for end-stage liver disease, *AFP* alpha-fetoprotein, *TTV* total tumor volume

^a^Milan criteria: 1 tumor ≤ 5 cm or 2–3 tumors ≤ 3 cm

^b^Metroticket 2.0 criteria: sum of the size of the largest tumor in centimeters ≤ 4 and AFP ≤ 1000 ng/mL or sum of the size of the largest tumor in centimeters ≤ 5 and AFP ≤ 400 ng/mL or sum of the size of the largest tumor in centimeters ≤ 7 and AFP ≤ 200 ng/mL

^c^TTV/AFP criteria: TTV ≤ 115 cm^3^ and AFP ≤ 400 ng/mL

^d^Warsaw criteria: within Milan criteria or within either University of California, San Francisco criteria or up-to-7 criteria with AFP ≤ 100 ng/mL

During a median follow-up period of 41.3 months, 34 patients experienced tumor recurrence, and 50 patients died. The median time to recurrence was 14.6 months. Overall, the cumulative 5-year incidence of recurrence was 17% for the patients without neoadjuvant treatment, 28.5% for the patients who had neoadjuvant treatment without CPR, and 14% for the patients who had neoadjuvant treatment with CPR (*p* = 0.157). Accordingly, no significant associations between neoadjuvant treatment with CPR (exp[β] = 0.78; 95% CI, 0.23–2.70; *p* = 0.700) or neoadjuvant treatment without CPR (exp[β] = 1.79; 95% CI, 0.87–3.70; *p* = 0.110) and post-transplantation risk of recurrence were identified.

The significant predictors of tumor recurrence were tumor number (*p* < 0.001), AFP (*p* = 0.001), Metroticket 2.0 (*p* < 0.001), AFP model (*p* < 0.001), TTV/AFP criteria (*p* < 0.001), and Warsaw criteria (*p* < 0.001). After adjustment for their effects in multivariable analyses, a complete response to neoadjuvant treatment was not found to be associated with a decreased risk of tumor recurrence (Table 2). Furthermore, all regression coefficients were positive, pointing toward nonsignificantly increased risk of tumor recurrence after adjustment for these confounders.

**Table 2:** Results of multivariable competing risk regression analyses of risk factors for tumor recurrence including morphologic variables combined with AFP having no response to neoadjuvant treatment and corresponding models with response to neoadjuvant treatment

Models	Exp(β)	95% CI for exp(β)	*p* Value	BIC	∆BIC^a^
Model 1A				331.86	10.19
No. of tumors	1.23	1.10–1.37	< 0.001		
Size of largest tumor	1.01	0.99–1.03	0.240		
AFP	1.85	1.28–2.67	0.001		
Model 1B				342.05	
No. of tumors	1.23	1.10–1.38	<0.001		
Size of largest tumor	1.01	0.99–1.03	0.350		
AFP	1.87	1.28–2.74	0.001		
Neoadjuvant treatment					
No	Reference				
Yes, with no CPR	1.33	0.61–2.90	0.470		
Yes, with CPR	1.34	0.37–4.85	0.660		
Model 2A				324.49	9.56
Metroticket 2.0	1.84	1.40–2.41	< 0.001		
Model 2B				334.05	
Metroticket 2.0	1.88	1.37–2.57	< 0.001		
Neoadjuvant treatment					
No	Reference				
Yes, with no CPR	1.27	0.58–2.75	0.550		
Yes, with CPR	2.10	0.54–8.19	0.280		
Model 3A				325.58	10.27
AFP model	1.55	1.30–1.84	< 0.001		
Model 3B				335.85	
AFP model	1.52	1.25–1.85	<0.001		
Neoadjuvant treatment					
No	Reference				
Yes, with no CPR	1.30	0.59–2.88	0.510		
Yes, with CPR	1.03	0.31–3.38	0.960		
Model 4A				326.78	8.85
Beyond TTV/AFP criteria	5.82	2.96–11.40	< 0.001		
Model 4B				335.63	
Beyond TTV/AFP criteria	5.52	2.73–11.16	< 0.001		
Neoadjuvant treatment:					
No	Reference				
Yes, with no CPR	1.65	0.78–3.48	0.190		
Yes, with CPR	1.09	0.33–3.58	0.880		
Model 5A				331.64	10.28
Beyond Warsaw criteria	4.26	2.19–8.28	< 0.001		
Model 5B				341.93	
Beyond Warsaw criteria	4.05	1.81–9.09	0.001		
Neoadjuvant treatment					
No	Reference				
Yes, with no CPR	1.31	0.58–2.95	0.520		
Yes, with CPR	1.21	0.35–4.13	0.760		

Competing risk regression coefficients were calculated for 1 tumor increase for the number of tumors, 1-mm increase for tumor size, 1-log_10_(ng/mL) increase for AFP, 1-point increase for Metroticket 2.0 model and AFP model

*AFP* alpha-fetoprotein, *β* competing risk regression coefficient, *CI* confidence interval, *BIC* Bayesian information criterion, *CPR* complete pathologic response, *TTV* total tumor volume

^a^∆BIC = BIC(model B) − BIC (model A)

Finally, the models incorporating a response to neoadjuvant treatment had a BIC 8.85 to 10.28 higher than the corresponding models without a response to neoadjuvant treatment, pointing toward their worse performance. No benefits regarding improvement of patient selection were observed in terms of NRI when a response to neoadjuvant treatment was added to the AFP model, the Metroticket 2.0 model, the TTV/AFP criteria, the Warsaw criteria, or simply to tumor size, number, and AFP (Table 3).

**Table 3:** Net reclassification improvement (NRI) analyses of adding neoadjuvant treatment response to tumor burden and alpha-fetoprotein in selection of hepatocellular patients for liver transplantation with respect to tumor recurrence prediction

Baseline model	NRI for modification of baseline model by adding response to neoadjuvant treatment			
	NRI	95% CI for NRI	*Z*	*p* value
Model 1	− 0.018	− 0.067 to 0.032	− 0.055	0.478
Tumor number				
Tumor size				
Alpha-fetoprotein				
Model 2	0.056	− 0.021 to 0.133	0.115	0.546
AFP model				
Model 3	− 0.005	− 0.061 to 0.052	− 0.013	0.495
Metroticket 2.0 model				
Model 4	–^a^	–	–	–
TTV/AFP criteria^a^				
Model 5	0.038	–0.079 to 0.155	0.051	0.520
Warsaw criteria				

*CI* confidence interval, *AFP* alpha-fetoprotein, *TTV* total tumor volume

^a^Response to neoadjuvant treatment added to TTV/AFP criteria did not reclassify any patient

Considering the general low-risk profile of the patients achieving CPR, a series a subgroup analyses of the patients within the Milan criteria and selected criteria based on a combination of morphologic and biologic factors were performed (Table 4). These showed a uniform nonsignificantly higher incidence of recurrence among the patients who had neoadjuvant treatment with CPR and a significantly increased incidence of recurrence among the patients who underwent neoadjuvant treatment without CPR within the Milan criteria (*p* = 0.016), the Warsaw criteria (*p* = 0.007), or the AFP model not exceeding 2 points (*p* = 0.021).

**Table 4:** Cumulative incidences of recurrence and Kaplan–Meier overall survival estimates 5 years after liver transplantation for hepatocellular carcinoma for patients in selected low-risk subgroups based on response to neoadjuvant treatment

Outcome measure	Subgroup	No neoadjuvant treatment (reference)	Neoadjuvant treatment without CPR		Neoadjuvant treatment with CPR		Overall *p* value
		5-Year estimate (%)	5-Year estimate (%)	*p* value^a^	5-Year estimate (%)	*p* value^a^	
Incidence of recurrence	Within Milan criteria	5.0	22.2	0.016	13.3	0.290	0.027
Incidence of recurrence	AFP model ≤ 2	7.4	23.8	0.021	10.4	0.740	0.033
Incidence of recurrence	Within Metroticket 2.0 criteria	9.2	21.1	0.110	14.0	0.580	0.257
Incidence of recurrence	Within TTV/AFP criteria	9.7	23.6	0.120	10.4	0.940	0.197
Incidence of recurrence	Within Warsaw criteria	4.5	25.4	0.007	14.0	0.130	0.008
Overall survival	Within Milan criteria	83.6	78.5	0.903	68.0	0.249	0.634
Overall survival	AFP model ≤ 2	82.9	70.6	0.263	72.1	0.359	0.541
Overall survival	Within Metroticket 2.0 criteria	82.0	70.2	0.298	73.2	0.474	0.602
Overall survival	Within TTV/AFP criteria	82.4	69.3	0.221	72.1	0.383	0.507
Overall survival	Within Warsaw criteria	84.0	75.0	0.655	73.2	0.395	0.837

*CPR* complete pathologic response, *AFP* alpha-fetoprotein, *TTV* total tumor volume

^a^Relative to patients without neoadjuvant treatment

The overall survival rate was 79.5% at 5 years for the patients without neoadjuvant treatment, 66.1% for those who underwent neoadjuvant treatment without CPR, and 73.2% for those who underwent neoadjuvant treatment with CPR (*p* = 0.363). Accordingly, neither the presence (HR, 1.21; 95% CI, 0.50–2.89; *p* = 0.938) nor the absence (HR, 1.55; 95% CI, 0.85–2.84; *p* = 0.249) of CPR was a significant prognostic factor for overall survival. The AFP model (*p* = 0.022), the TTV/AFP criteria (*p* = 0.013), and the Warsaw criteria (*p* = 0.001) were significantly associated with overall survival, whereas the Metroticket 2.0 model did not reach significance (*p* = 0.109). After adjustment for their effects, CPR was not associated with the risk of tumor recurrence, with the absolute HRs ranging from 1.32 to 1.63 (Table 5).

**Table 5:** Results from multivariable analyses of risk factors for worse overall survival including morphologic variables combined with AFP having no response to neoadjuvant treatment and corresponding models with response to neoadjuvant treatment

Models	HR^a^	95% CI for HR	*p* value	BIC	∆BIC^b^
Model 1A				514.67	6.09
Metroticket 2.0	1.19	0.96–1.47	0.109		
Model 1B				520.76	
Metroticket 2.0	1.20	0.94–1.52	0.144		
Neoadjuvant treatment					
No	Reference				
Yes, with no CPR	1.44	0.77–2.66	0.676		
Yes, with CPR	1.56	0.60–4.01	0.573		
Model 2A				512.36	6.48
AFP model	1.19	1.03–1.38	0.022		
Model 2B				518.84	
AFP model	1.18	1.01–1.38	0.039		
Neoadjuvant treatment					
No	Reference				
Yes, with no CPR	1.42	0.77–2.63	0.493		
Yes, with CPR	1.32	0.55–3.19	0.805		
Model 3A				511.65	6.10
Beyond TTV/AFP criteria	2.24	1.19–4.23	0.013		
Model 3B				517.75	
Beyond TTV/AFP criteria	2.19	1.15–4.19	0.017		
Neoadjuvant treatment					
No	Reference				
Yes, with no CPR	1.49	0.81–2.74	0.403		
Yes, with CPR	1.34	0.55–3.23	0.826		
Model 4A				507.73	6.29
Beyond Warsaw criteria	2.51	1.44–4.40	0.001		
Model 4B				514.02	
Beyond Warsaw criteria	2.59	1.41–4.75	0.002		
Neoadjuvant treatment					
No	Reference				
Yes, with no CPR	1.35	0.73–2.50	0.858		
Yes, with CPR	1.63	0.66–4.05	0.440		

*AFP* alpha-fetoprotein, *HR* hazard ratio, *CI* confidence interval, *BIC* Bayesian information criterion, *CPR* complete pathological response, *TTV* total tumor volume

^a^Hazard ratios were calculated for 1 point increase for the Metroticket 2.0 model and the AFP model

^a^∆BIC = BIC (model B) − BIC (model A)

Comparison of BIC values also did not support inclusion of a response to neoadjuvant treatment with the existing criteria in terms of predicting overall survival. For the patients within Milan criteria, the AFP model not exceeding 2 points, the Metroticket 2.0 criteria, the TTV/AFP criteria, and the Warsaw criteria including both CPR and lack of CPR were associated with nonsignificantly lower overall survival rates (Table 4).

**Fig. 1:**
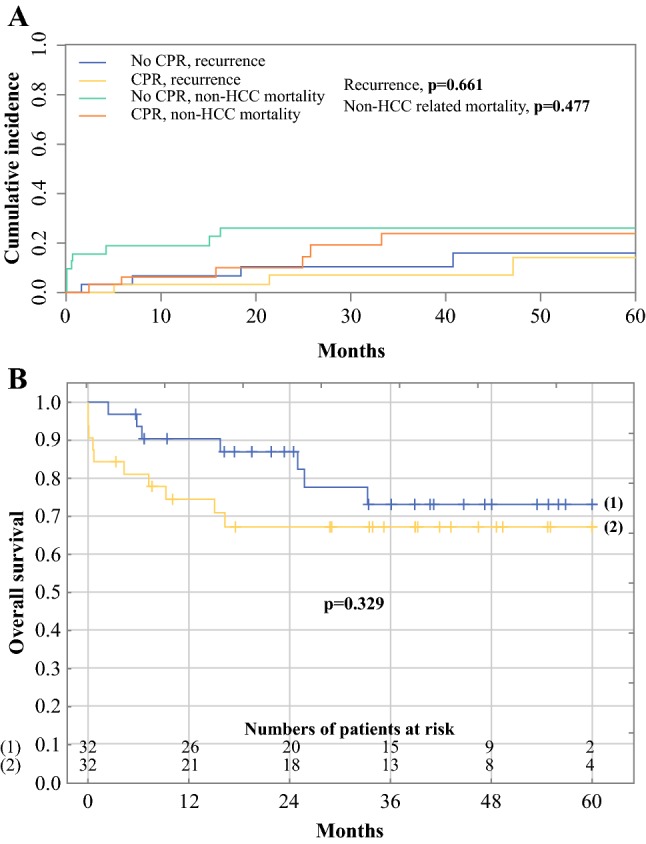
Comparison of propensity score-matched patients with and without a complete pathologic response (CPR) after neoadjuvant therapy with respect to **a** competing risk-adjusted cumulative incidence of tumor recurrence and non-hepatocellular carcinoma (HCC)-related mortality, and **b** overall survival after liver transplantation (blue line—patients with CPR; yellow line—patients without CPR)

The patients achieving CPR were matched in a 1:1 ratio with the patients who did not achieve CPR using a propensity score based on tumor number, tumor size, and AFP. In this matched cohort, the 5-year cumulative incidence of recurrence was 14% for the patients who had neoadjuvant treatment and achieved CPR compared with 15.9% for those who did not achieve CPR (*p* = 0.661; Fig. 1a). The corresponding incidence rates for non–HCC-related mortality adjusted for the competing risk of recurrence were 23.7% and 26.0%, respectively (*p* = 0.477, Fig. 1a). The overall survival rate was 73.2% for the patients who achieved CPR and 67.4% for the patients without CPR (*p* = 0.329, Fig. 1b).

## Discussion

A CPR to neoadjuvant treatment is commonly reported as an extremely favorable factor associated with minimal risk of HCC recurrence after liver transplantation.^19^,^20^,^28^ The results of this study indicate that the favorable outcomes for HCC patients who achieve CPR after neoadjuvant treatment are due to their favorable baseline profile of recurrence risk rather than the prognostic impact of the response. According to performed analyses adjusted for the confounding effects of differences in baseline characteristics, assessment of CPR to neoadjuvant treatment does not improve the capability of selection criteria combining morphologic features with AFP to predict tumor recurrence.

In general, CPR was associated with post-transplantation outcomes similar to those for the neoadjuvant treatment-naïve patients and nonsignificantly worse than for the patients without CPR. Irrespective of statistical significance, such absolute differences appear to be in line with differences in patients’ baseline risk factors for tumor recurrence, with the lowest risk expected for patients with CPR followed by treatment-naïve patients and those without CPR after neoadjuvant therapy. Notably, the favorable characteristics of patients who achieve CPR are uniformly reported in other studies, with predictors of response often being well-established predictors of tumor recurrence.^20^,^28^,^29^

Notably, none of the performed multivariable analyses pointed toward even a nonsignificant positive effect of CPR in terms of decreased risk for tumor recurrence. After adjustment for the confounding effects of different baseline characteristics through propensity score matching, the patients who achieved and those did not achieve CPR were observed to have almost identical post-transplantation outcomes. Nevertheless, the results do not undermine the rationale for performing neoadjuvant therapy to achieve CPR because it is associated with favorable changes in other risk factors and thus may limit the risk of tumor recurrence. For instance, previous studies have shown that lowering the AFP level after neoadjuvant treatment remarkably limits the risk of post-transplantation recurrence and obviously may enable liver transplantation if values drop below the cutoff for particular criteria.^30^–^32^

Although the lack of a prognostic role for CPR to neoadjuvant treatment was a rather unexpected finding, a previous study by Kang et al.^21^ showed that patients who underwent either liver resection or transplantation after neoadjuvant treatment had a significantly higher risk of recurrence than low-risk treatment-naïve patients despite achievement of CPR. Importantly, low-risk control groups in that previous study comprised patients with single tumors smaller than 2 cm eligible for liver resection and up to two tumors smaller than 2 cm eligible for liver transplantation. In that setting, comparison of baseline characteristics showed that the patients with CPR were in fact at increased baseline risk of tumor recurrence, as indicated by a higher AFP level and a larger tumor size.

The results of this study confirm that a combination of morphologic features with AFP enables selection of HCC patients for liver transplantation, in line with numerous previous studies.^6^–^10^ The obtained results show that the addition of another biologic criterion represented by a complete response to neoadjuvant treatment not only does not improve the predictive efficacy of those models, but also is associated with their worse performance, a finding reported for the first time in the literature. Notably, none of the previous studies demonstrated that predictive ability improved when a response to neoadjuvant treatment was added to the existing morphologic and biologic criteria. Importantly, the last pre-transplantation patient characteristics were used for patient matching and adjustment of the effects from CPR because these are particularly associated with both the response to treatment and the risk of recurrence.^20^,^30^–^32^

Interestingly, neoadjuvant treatment without CPR was associated with significantly higher recurrence rates for several low-risk groups of patients. These included patients within the Milan criteria, those with an AFP model not exceeding 2 points, and those within the Warsaw criteria. The unexpectedly high rates of tumor recurrence for these generally low-risk patients receiving neoadjuvant treatment correspond to the results of a recent large-scale study by the U.S. Multicenter HCC Transplant Consortium, in which neoadjuvant treatment without CPR was independently associated with higher risk of recurrence.^30^

Because the current study aimed to investigate the prognostic effect of CPR, the results are insufficient to support the conclusion that neoadjuvant therapy may potentially increase the risk of post-transplantation recurrence for low-risk HCC patients. The potential reasons for such an association were, however, provided in a recent study by Xu et al.^18^, in which neoadjuvant therapy-induced necrosis was associated with increased lymphangiogenesis and increased risk of post-transplantation lymphatic metastases. Therefore, this is another report on the potential negative effect of neoadjuvant therapy and may be considered as an argument for more cautious selection of low-risk patients for pre-transplantation bridging.

Although the results of the current study contradict the prognostic relevance of CPR and the rationale for its evaluation during pre-transplantation assessment, they do not undermine the role of general radiologic assessment of response to neoadjuvant treatment before liver transplantation. Progression of the disease during the pre-transplantation period is consistently reported as a negative prognostic factor for post-transplantation recurrence, and the current study did not reevaluate its prognostic impact.^19^,^33^–^35^ On the contrary, assessments of the optimal method for predicting CPR seem unnecessary in the context of the presented results.

The current study had several limitations. First, its retrospective character was associated with all the drawbacks inherent to retrospective studies. Second, the study did not include any data on tumor progression in the pre-transplantation period. However, it aimed to evaluate the prognostic significance of CPR and not a response to treatment in general. Furthermore, there were no data on radiologic assessment of response to treatment, yet pathologic evaluation is the gold standard for evaluating a complete response to treatment.^17^,^36^ Nevertheless, the results indicating that CPR lacks prognostic significance should not be extrapolated to mean that radiologic assessment of a treatment response lacks prognostic significance.

In conclusion, CPR after neoadjuvant treatment does not have prognostic significance in liver transplantation for HCC when adjusted for the effects of differences in baseline characteristics. Patients with low-risk HCCs should cautiously be selected for bridging therapies due to the potential increase of the risk for post-transplantation tumor recurrence.
